# Fabrication, Characterization, and Transcriptomic Analysis of Oregano Essential Oil Liposomes for Enhanced Antibacterial Activity and Sustained Release

**DOI:** 10.3390/foods15010157

**Published:** 2026-01-03

**Authors:** Zhuo Wang, Yuanxin Bao, Jianguo Qiu, Shanshan Li, Hong Chen, Cheng Li

**Affiliations:** 1 College of Food Science, Sichuan Agricultural University, Yaan 625014, China; 2020118003@stu.sicau.edu.cn (Z.W.); bao19162805972@163.com (Y.B.); ssli1205@163.com (S.L.); 2 School of Food Science and Technology, Jiangnan University, Wuxi 214122, China; 7250112119@stu.jiangnan.edu.cn; 3 Key Laboratory of Agricultural Product Processing and Nutrition Health (Co-Construction by Ministry and Province), Ministry of Agriculture and Rural Affairs, Yaan 625014, China

**Keywords:** oregano essential oil, liposomes, stability, antibacterial mechanism, transcriptomics, sustained release

## Abstract

This study prepared oregano essential oil-loaded liposomes (OEO-Lip) and systematically evaluated their physicochemical properties, stability, and antioxidant/antibacterial activities, along with the underlying mechanisms. Characterization revealed OEO-Lip exhibited a unilamellar vesicle structure with a particle size of approximately 190 nm, uniform dispersion (PDI = 0.183), a high zeta potential (−39.8 mV), and an encapsulation efficiency of 77.52%. Analyses by FT-IR, XRD, and DSC confirmed the successful encapsulation of OEO within the liposomes. Hydrogen bonding interactions with phospholipid components promoted the formation of a more ordered crystalline structure, thereby enhancing thermal stability. Storage stability tests demonstrated that OEO-Lip stored at 4 °C for 30 days exhibited significantly superior physicochemical properties compared to samples stored at 25 °C. Furthermore, liposomal encapsulation effectively preserved the antioxidant activity of OEO. Antimicrobial studies revealed that OEO-Lip exerted stronger and more sustained inhibitory effects against *Escherichia coli* and *Staphylococcus aureus* than free OEO, primarily by disrupting bacterial membrane integrity and inducing the leakage of ions and intracellular contents. Transcriptomic analysis further indicated that OEO-Lip exerts synergistic antibacterial effects by downregulating genes associated with phospholipid synthesis and nutrient transport while concurrently interfering with multiple pathways, including quorum sensing and energy metabolism. Release experiments indicated that OEO-Lip displays both burst and sustained release characteristics. In summary, OEO-Lip serves as an efficient delivery system that significantly enhances the stability and antibacterial efficacy of OEO, demonstrating considerable potential for application in food preservation.

## 1. Introduction

Foodborne illness remains a significant global public health challenge [1]. Foodborne pathogens, such as Gram-negative *Escherichia coli* [2] and Gram-positive *Staphylococcus aureus* [3], are major causative agents of food spoilage and safety incidents. These bacteria exhibit substantial differences in cell wall structure, membrane composition, and metabolic regulatory networks. *S. aureus* possesses a thick peptidoglycan layer [4], whereas *E. coli* has an outer membrane structure composed of peptidoglycan and lipopolysaccharide (LPS) [5]. These structural differences directly influence membrane fluidity and susceptibility to hydrophobic antimicrobials, underscoring the importance of developing targeted control strategies [6]. Although synthetic chemical preservatives effectively inhibit microbial growth, their potential health risks and environmental impact have raised concerns. Consequently, consumer demand for clean-label and natural preservative solutions is increasing.

Oregano essential oil (OEO), a widely used natural antimicrobial agent, is a yellow, clear oily liquid extracted from the oregano plant. Its main active components, carvacrol and thymol, feature a phenolic ring structure formed by two isoprene units and three functional group substituents, which confer antibacterial and antioxidant activities [7]. Studies indicate that OEO inhibits various foodborne pathogens, including *E. coli* and *S. aureus* [8]. The hydrophobic active components in OEO, such as carvacrol, can directly penetrate the thick peptidoglycan layer of *S. aureus* to reach the cell membrane [9]. For *E. coli*, they must first traverse the hydrophobic barrier of the LPS-containing outer membrane. Research shows that carvacrol can disrupt LPS structure, breaching this defense and contributing to OEO’s broad-spectrum antimicrobial activity [10]. However, the volatility, water insolubility, and chemical instability of OEO severely limit its practical application in food systems [11]. To address these limitations, researchers have explored various nanocarrier systems, such as nanoemulsions [12] and cyclodextrin inclusion complexes [13]. For instance, OEO-loaded nanoemulsions stabilized by whey protein isolate showed improved aqueous solubility but poor storage stability at 4 °C for more than 2 weeks [12]. Cyclodextrin-OEO complexes enhanced thermal stability but exhibited low encapsulation efficiency due to mismatched cavity size [13], highlighting the need for more suitable delivery systems.

Liposomes, as nanodelivery systems with excellent biocompatibility and tunable release characteristics, offer a viable pathway to enhance the stability, bioavailability, and targeted delivery efficiency of hydrophobic active substances like OEO [14]. Their amphiphilic structure enables the encapsulation of essential oils within the phospholipid bilayer, isolating the food from harmful storage conditions while providing controlled release of active components to extend freshness [15]. Liposomes are vesicular systems that are typically composed of phospholipids and cholesterol, mimicking biological membranes. They can be unilamellar or multilamellar and are widely used in food applications due to their biocompatibility, targeting potential, sustained-release properties, and ability to deliver hydrophobic compounds [16]. The hydrophilic phospholipid heads form an ordered interfacial network in the aqueous phase, while the hydrophobic fatty acid tails constitute a liquid-ordered core matrix [17]. Incorporation of essential oil molecules can cause local membrane curvature deformation, prompting adjacent lipid molecules to reorganize and maintain structural integrity [18].

The traditional thin-film dispersion method is commonly used for liposome preparation. This method initially forms a uniform lipid film, ensuring sufficient encapsulation of hydrophobic actives within the bilayer [19]. Although effective and straightforward, liposomes prepared solely by this method often exhibit larger particle sizes and broad size distribution [20]. Combining the thin-film dispersion method with high-pressure microfluidization can address these limitations. The extreme shear forces generated during microfluidization disrupt and reassemble large, multilamellar liposomes, promoting more orderly phospholipid packing [21]. This process facilitates the formation of a more compact and dense lipid bilayer, ultimately yielding unilamellar liposomes with a controlled size of 100–200 nm and a narrow size distribution [22]. However, critical questions remain regarding the nanoarchitecture and storage stability of OEO-Lip prepared via this combined technique, its sustained-release inhibitory efficacy and mechanism against *E. coli* and *S. aureus*, and the release kinetics of OEO within food simulants. Notably, high-pressure microfluidization technology has been applied in the preparation of camellia seed oil liposomes, achieving enhanced stability and sustained-release performance [23]. Yet, research on its application to OEO liposome fabrication remains scarce, especially regarding the optimization of process parameters tailored to the unique phenolic components of OEO. However, several critical issues pertaining to OEO liposomes prepared via this combined technique remain to be elucidated: their nanostructure, storage stability, sustained-release inhibitory efficacy and underlying mechanisms against *E. coli* and *S. aureus*, as well as release kinetics in food simulants.

Therefore, this study aims to fabricate oregano essential oil liposomes (OEO-Lip) via the combined thin-film dispersion and high-pressure microfluidization approach, and to address the inherent limitations associated with single fabrication methods. We will systematically evaluate its physicochemical properties, storage stability, and antioxidant activity, with a focus on its antibacterial effects and mechanisms against *E. coli* and *S. aureus*. By integrating phenotypic analyses (e.g., membrane permeability, intracellular content leakage) with transcriptomic (RNA-Seq) profiling, we seek to elucidate the molecular targets and regulatory networks affected by OEO-Lip at the gene expression level. Furthermore, the release behaviour of OEO-Lip in different food simulants will be assessed to provide a theoretical basis for its controlled release and targeted application in food preservation.

## 2. Materials and Methods

### 2.1. Materials

Oregano essential oil (OEO) was purchased from Shanghai Yuanye Bio-Technology Co., Ltd. (Shanghai, China). Egg yolk lecithin (purity > 90%), cholesterol (purity > 95%), and Tween 80 were obtained from Sangon Biotech (Shanghai) Co., Ltd. (Shanghai, China). *Staphylococcus aureus* and *Escherichia coli* were provided by the Microbiology Laboratory of the College of Food Science, Sichuan Agricultural University. All other chemicals used were of analytical grade unless otherwise specified.

### 2.2. Preparation of Oregano Essential Oil-Liposomes (OEO-Lip)

OEO-Lip was prepared using the thin-film dispersion method coupled with high-pressure microfluidization. The preparation conditions were optimized to achieve liposomes with the highest encapsulation efficiency (EE), using the polydispersity index (PDI), mean particle size, and EE as evaluation indices. The optimization was performed through single-factor experiments followed by response surface methodology (RSM), with detailed methods and results provided in the Supplementary Materials Figures S1 and S2. The liposomes were subsequently prepared according to the optimized parameters. Briefly, 400 mg of egg yolk lecithin and 101.01 mg of cholesterol (mass ratio of 3.96:1), along with 10 mg of Tween 80, were accurately weighed. An OEO concentration of 1.82 mg/mL was used. The mixture was dissolved in 20 mL of anhydrous ethanol. The organic solvent was then removed using a rotary evaporator (RE-200B, Jiapeng Technology Co., Ltd., Shanghai, China) operating at 40 rpm, and 44.34 °C until a thin, uniform lipid film formed on the inner wall of the eggplant-shaped flask. Subsequently, 26 mL of phosphate-buffered saline (PBS, pH 7.0) was added to hydrate the lipid film under bath sonication (KQ-300DE, Kun Shan Ultrasonic Instruments Co., Ltd., Shanghai, China). Finally, the suspension was homogenized for 3 cycles at 120 MPa using a high-pressure microfluidizer (MF110P-A, Antuos Nanotechnology Co., Ltd., Suzhou, China). The resulting OEO-Lip dispersion was allowed to stand for further use.

### 2.3. Determination of Entrapment Efficiency (EE)

A standard OEO–ethanol solution (1 mg/mL) was prepared, and the maximum absorption wavelength of OEO (measured via UV-1800PC, Mapada Instruments Co., Ltd., Shanghai, China) was determined to be 278 nm by full-wavelength scanning. A standard stock solution (1 mg/mL) was diluted with anhydrous ethanol to create a series of standard solutions (20, 40, 50, 60, 80, 100 μg/mL). A certain amount of analytically pure OEO was accurately weighed using an analytical balance. An appropriate volume of anhydrous ethanol was added to dissolve the OEO completely, followed by ultrasonic treatment for 10 min to ensure uniform dispersion. The mixture was then transferred to a volumetric flask and diluted to the mark with anhydrous ethanol to obtain the 1 mg/mL standard stock solution. The stock solution was stored at 4 °C in the dark for subsequent use. The absorbance of these solutions was measured at 278 nm to establish a standard curve: y = 0.0066x − 0.0095 (R^2^ = 0.9994). To determine the total OEO concentration (C_0_), 1 mL of the OEO-Lip dispersion was diluted to 25 mL with anhydrous ethanol and subjected to ultrasonication to disrupt the liposomes. The absorbance of this solution was measured at 278 nm, using blank liposomes (Lip) as a control, and C_0_ was calculated from the standard curve. To determine the free OEO concentration (C_1_), 1 mL of the OEO-Lip dispersion was mixed with 4 mL of n-hexane, vortexed thoroughly, and centrifuged at 8000× *g* for 30 min. This step was repeated several times. The resulting organic phases were combined, diluted to 25 mL with anhydrous ethanol, and their absorbance was measured to calculate C_1_. The encapsulation efficiency (EE) was calculated using the following Equation (1):

(1) $$EE\%=(1-C_{1}/C_{0})\times 100\%$$

where: EE is the encapsulation efficiency (%); C_1_ is the concentration of free OEO; C_0_ is the total OEO concentration in the liposomal dispersion.

### 2.4. Determination of Particle Size and Zeta (ζ)-Potential

The sample was diluted fivefold with distilled water for subsequent analysis. The particle size, ζ-potential, and polydispersity index (PDI) were measured using a Malvern Zetasizer Nano-ZS (Malvern Instruments Ltd., Worcestershire, UK).

### 2.5. Structural Characterization of OEO-Lip

#### 2.5.1. Transmission Electron Microscopy (TEM)

A 5 μL aliquot of the sample was placed onto a carbon-coated copper grid for 3 min. The excess liquid was wicked away with filter paper, and then 2% phosphotungstic acid (5 μL) was added; this was allowed to stand for 3 min. The remaining stain was removed, and the grid was allowed to air-dry at room temperature before imaging the morphology via transmission electron microscopy (TEM) (Regulus8100, Hitachi Ltd., Tokyo, Japan).

#### 2.5.2. Fourier Transform Infrared (FT-IR) Spectroscopy

FT-IR spectra were recorded using a Nicolet IS10 spectrometer (Thermo Fisher Scientific Inc., Waltham, MA, USA) in the range of 500–4000 cm^−1^ using the KBr pellet method. Spectra of free OEO, blank liposomes (Lip), OEO-Lip, and their physical mixture (PM) were acquired. The PM was prepared by uniformly mixing egg yolk lecithin (400 mg), cholesterol (100 mg), Tween 80 (40 mg), and OEO (36 mg) at room temperature.

#### 2.5.3. Differential Scanning Calorimetry (DSC)

Thermal behavior was analyzed using DSC (Q2000, TA Instruments, New Castle, DE, USA). Approximately 5 mg of lyophilized samples (Lip, OEO, OEO-Lip) were sealed in aluminum pans, with an empty pan used as a reference. The analysis was performed under a nitrogen atmosphere (purge rate: 30 mL/min) by heating from 20 to 300 °C at a rate of 20 °C/min.

#### 2.5.4. Thermogravimetric Analysis (TGA)

TGA was conducted to monitor mass loss. Samples (Lip, OEO, OEO-Lip, PM) were heated from room temperature to 800 °C at a heating rate of 10 °C/min using a TGA Q500 analyzer (TA Instruments, New Castle, DE, USA).

#### 2.5.5. X-Ray Diffraction (XRD)

XRD patterns of Lip, OEO, OEO-Lip, and PM were recorded using a DX-2700B diffractometer (Dandong, China). The analysis was performed using Cu Kα radiation at 30 kV and 10 mA. The diffraction patterns were recorded over a 2θ range of 5–60° with a scanning speed of 3°/min.

### 2.6. Storage Stability of OEO-Lip

The EE, particle size, and ζ-potential of OEO-Lip during storage were determined using the methods described in Section 2.3 and Section 2.4. For pH determination, OEO-Lip was diluted with deionized water at a 1:1 ratio, and the pH was measured using a pH meter (PHS-3C, Shanghai Leici Instrument Factory, Shanghai, China).

### 2.7. Retention Rate of OEO in OEO-Lip During Storage

OEO-Lip samples were stored under two different conditions, 4 °C (refrigeration) and 25 °C (room temperature), for a period of 30 days. Samples were collected at predetermined time intervals (1, 2, 3, 6, 9, 12, 18, 24, and 30 days) for subsequent analysis. The retention rate of OEO in OEO-Lip was determined by following the same procedure for C_0_ determination in Section 2.3 (1 mL OEO-Lip diluted to 25 mL with anhydrous ethanol, followed by ultrasonic demulsification). Using Lip as a control, the absorbance at 278 nm was measured to calculate the total mass of OEO in OEO-Lip via the standard curve. The retention rate was calculated as:

(2) $$Retention\;rate\;(\%)=(m_{n}/m_{0})\times 100\%$$

where, m_0_ is the total mass of OEO in OEO-Lip on day 0, and m_n_ is the total mass of OEO in OEO-Lip on day n.

### 2.8. Determination of Malondialdehyde (MDA) Content

The MDA content in the liposomes was determined by the thiobarbituric acid (TBA) method during storage. Briefly, 1.0 mL of the OEO-Lip dispersion was transferred into a 10 mL graduated test tube. Then, 5 mL of a TCA-TBA-HCl mixture solution (containing trichloroacetic acid, thiobarbituric acid, and hydrochloric acid) was added and mixed thoroughly. The mixture was heated in a boiling water bath for 30 min, cooled to room temperature, and subsequently made up to the mark with the TCA-TBA-HCl solution. After centrifugation at 8000× *g* for 10 min, the absorbance of the supernatant was measured at 535 nm, using the TCA-TBA-HCl solution as a blank.

### 2.9. Determination of Antioxidant Activity of OEO-Lip

#### 2.9.1. DPPH Radical Scavenging Assay

The DPPH radical scavenging activity was determined as follows: OEO-Lip was mixed with a DPPH-ethanol solution. After homogenization, the mixture was incubated in the dark for 30 min, followed by centrifugation at 8000× *g* for 10 min. The absorbance of the supernatant was measured at 525 nm. The DPPH radical scavenging rate was calculated according to the following Formula (3):

(3) $$DPPH\;radical\;scavenging\;activity\;(\%)=(1-\frac{A_{2}-A_{1}}{A_{0}})\times 100\%$$

where A_0_, A_1_, and A_2_ represent the absorbance values at 525 nm for the DPPH solution mixed with anhydrous ethanol, the sample solution mixed with anhydrous ethanol, and the sample solution mixed with the DPPH solution, respectively.

#### 2.9.2. ABTS^+^ Radical Scavenging Assay

The ABTS^+^ radical scavenging activity was assessed as follows: OEO-Lip was mixed with a pre-prepared ABTS^+^ radical stock solution, which had an initial absorbance of 0.70 ± 0.02 at 734 nm. The mixture was vortexed thoroughly and allowed to stand for 10 min at room temperature. It was then centrifuged at 8000× *g* for 10 min. The absorbance of the resulting supernatant was measured at 734 nm. The ABTS^+^ radical scavenging rate was calculated using the following Formula (4):

(4) $${ABTS}^{+}\;radical\;scavenging\;activity\;(\%)=(1-\frac{A_{2}-A_{1}}{A_{0}})\times 100\%$$

where A_0_, A_1_ and A_2_ represent the absorbance values at 734 nm for the ABTS solution mixed with PBS buffer solution, the sample solution mixed with PBS buffer solution, and the sample solution mixed with the ABTS solution, respectively.

#### 2.9.3. Hydroxyl Radical (•OH) Scavenging Assay

The •OH scavenging activity was determined as follows: 1 mL of OEO-Lip was mixed with 1 mL of 10 mmol/L FeSO_4_ and 1 mL of 8.8 mmol/L H_2_O_2_ to initiate the reaction. The reaction mixture was incubated in a water bath at 37 °C for 30 min. The absorbance was then measured at 510 nm against a distilled water blank. The •OH scavenging rate was calculated using the following Formula (5):

(5) $$•OH\;Scavenging\;Activity\;(\%)=(1-\frac{A_{2}-A_{1}}{A_{0}})\times 100\%$$

where A_0_ represents the absorbance of the reaction mixture without the sample (distilled water instead); A_1_ represents the absorbance of the sample solution without the reaction reagents; and A_2_ represents the absorbance of the complete reaction mixture containing the sample.

### 2.10. Determination of Antibacterial Activity of OEO-Lip

#### 2.10.1. Total Viable Count (TVC)

This study evaluated the sustained antibacterial effects of OEO-Lip against *Escherichia coli* (*E. coli*) and *Staphylococcus aureus* (*S. aureus*). Glycerol stocks of *E. coli* (ATCC 25922) and *S. aureus* (ATCC 25923) stored at −80 °C were retrieved, thawed rapidly at room temperature, and a small aliquot of the bacterial suspension was collected using a sterile inoculation loop in a laminar flow hood. The harvested bacterial suspension was inoculated onto nutrient agar (NA) plates via the streak plate method and incubated in an inverted position in a constant-temperature incubator. Single colonies with uniform morphology were selected from the NA plates, inoculated into Erlenmeyer flasks containing 50 mL of nutrient broth (NB) medium, and cultured in a constant-temperature shaking incubator until the bacteria entered the logarithmic growth phase. The activated bacterial suspensions of *E. coli* and *S. aureus* were serially diluted in NB medium and then adjusted to an initial concentration of 10^6^ CFU/mL. To these suspensions, 2 mL of either OEO-Lip or an OEO solution (prepared with 1% Tween 80 to match the OEO concentration) was added, resulting in a final volume fraction of 20%. All cultures were incubated at 37 °C with shaking. Samples were taken every 24 h for colony counting to determine the long-term antibacterial performance.

#### 2.10.2. Growth Curve Determination

After activating bacterial strains to the logarithmic phase, they were inoculated into sterile test tubes containing NB medium and diluted to 10^6^ CFU/mL. OEO-Lip was added at concentrations of 20% and 40%, while an equal volume of sterile water was added as a blank control. The cultures were incubated at 37 °C, and the OD_600_ was measured every hour. Each group was set up with three replicates, and the growth curves were plotted based on the data.

#### 2.10.3. Scanning Electron Microscopy (SEM)

Bacterial suspensions (10^6^ CFU/mL) were treated with 20% (*v*/*v*) OEO-Lip and incubated at 37 °C for 6 h, using PBS as a blank control. After incubation, the samples were centrifuged at 8000× *g* for 10 min at 4 °C. The pellet was washed with PBS and fixed overnight with 2.5% glutaraldehyde at 4 °C. Subsequently, the bacteria were dehydrated using a graded ethanol series, dried in a desiccator, mounted on conductive tape, and sputter-coated with gold before observation using a ZEISS Sigma 300 SEM (Oberkochen, Germany).

#### 2.10.4. Cell Membrane Permeability

The activated bacterial cultures were centrifuged at 8000× *g* for 10 min. The supernatant was discarded, and the pellet was washed and resuspended in 20 mL of sterile saline. This washing step was repeated three times. The bacterial suspensions were then treated with OEO-Lip at working volume concentrations of 20% and 40%, using a group without OEO-Lip as a blank control. After thorough mixing, all samples were incubated at 37 °C. The electrical conductivity of the suspensions was measured at 0, 2, 4, 6, 8, and 10 h using a portable conductivity meter (712 Conductometer, Metrohm AG, Herisau, Switzerland). The experiment was performed in triplicate.

#### 2.10.5. Leakage of Bacterial RNA and Protein

OEO-Lip was added to bacterial suspensions (10^7^ CFU/mL) to achieve working concentrations of 20% and 40%, with a group without OEO-Lip serving as a blank control. After thorough mixing, the samples were incubated at 37 °C. At 0, 2, 4, 6, 8, and 10 h, aliquots were withdrawn and centrifuged at 8000× *g* for 5 min. The supernatant was collected, and nucleic acid content was determined by measuring OD_260_ nm using a NanoDrop spectrophotometer (NanoDrop 2000, Thermo Fisher Scientific Inc., Waltham, MA, USA). Protein content was measured using the Coomassie Brilliant Blue G-250 staining method. All experiments were performed in triplicate.

#### 2.10.6. Transcriptome Sequencing (RNA-Seq)

Activated suspensions of *S. aureus* and *E. coli* were inoculated into sterile NB medium. OEO-Lip was added to each culture at a final concentration of 20% (*v*/*v*), while an equal volume of sterile PBS was used as a blank control. The experimental groups were designated as follows: *S. aureus* control (Blank aureus), *S. aureus* treated with OEO-Lip (EG aureus), *E. coli* control (Blank coli), *E. coli* treated with OEO-Lip (EG coli). Each group included three biological replicates. After 6 h of incubation at 37 °C with shaking at 200 rpm, samples were collected, immediately flash-frozen in liquid nitrogen, and sent to Shanghai Majorbio Bio-pharm Technology Co., Ltd. (Shanghai, China) for transcriptome sequencing.

### 2.11. Release Profile of OEO-Lip in Food Simulants

The release behavior of the developed OEO-Lip was studied in three food simulants according to Commission Regulation (Annex III) EU No 10/2011. The release behavior of OEO-Lip was investigated using 10% ethanol, 50% ethanol, and PBS as food simulants. A 10 mL aliquot of the liposome suspension was placed in a dialysis bag (MWCO 14 kDa), which was then immersed in 500 mL of release medium and continuously shaken at 200 rpm and 25 °C (ZHWY-211B, Shanghai Zhichu Instrument Co., Ltd., Shanghai, China). Samples were taken at 1, 2, 3, 4, 5, 6, 7, 8, 12, 16, 24, and 36 h, with an equal volume of fresh medium replenished each time. The absorbance at 278 nm was measured to determine the OEO concentration. The cumulative release percentage (Q) of OEO at each time point was calculated using Equation (6). The release kinetics of OEO-Lip were further analyzed by fitting the data to appropriate mathematical models.

(6) $$Q=\frac{\left(C_{t}V_{0}+\sum_{n=1}^{t-1}C_{t-1}V\right)}{M_{0}}\times 100\%$$

where: Q represents cumulative release rate of OEO (%); C_t_ represents OEO concentration at the sampling time point; V_0_ represents volume of the release medium; V represents sample volume withdrawn; C_t−1_ represents OEO concentration at the previous sampling point; M_0_ represents total amount of OEO in the system.

### 2.12. Data Analysis

All results are expressed as the mean ± standard deviation. The data were primarily processed and statistically analyzed using Microsoft Excel 2019. Graphs were plotted using Origin 2021 software. Response surface methodology (RSM) optimization was performed with Design Expert software (version 8.06). Statistical significance of the data was analyzed with SPSS software (version 24.0), with a *p* < 0.05 considered statistically significant.

## 3. Results and Discussion

### 3.1. Characterization of OEO-Lip

#### 3.1.1. Microstructure, Particle Size, PDI, and Zeta Potential

**Figure 1:**
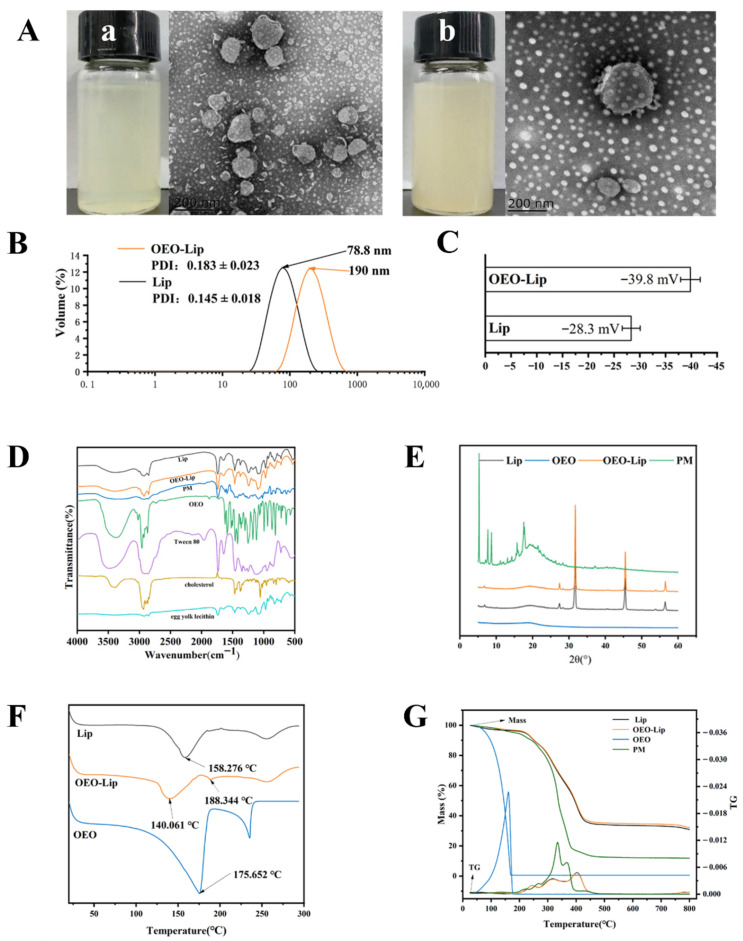
Structural characterization of OEO-Lip. (**A**) Morphology and TEM analysis of (**a**) Lip and (**b**) OEO-Lip. (**B**) Particle size and PDI. (**C**) Zeta potential. (**D**) FT-IR spectra. (**E**) XRD patterns. (**F**) DSC curves. (**G**) TGA curves.

The morphology of the optimized OEO-Lip formulation was examined by visual inspection and TEM. As shown in Figure 1A, the OEO-Lip appeared as a milky white, homogeneous emulsion. TEM images revealed bright, unilamellar vesicles with a unilocular structure, surrounded by dark edges, which indicated the successful encapsulation of oregano essential oil within the hydrophobic core of the liposomes. Subsequently, the particle size, PDI, and zeta potential of the OEO-Lip were characterized. Results presented in Figure 1B,C show that the mean particle size of Lip was 78.8 nm, while that of OEO-Lip increased to 190 nm. This size increase following OEO incorporation can be attributed to the integration of essential oil into the lipid bilayer, which reduces the cohesion of phospholipid molecules [24]. The PDI of OEO-Lip was measured as 0.183, which is below 0.3, indicating a homogeneous dispersion within the system. The zeta potential of blank Lip was −28.3 mV, whereas OEO-Lip exhibited a significantly higher negative charge of −39.8 mV. This strongly negative surface charge promotes electrostatic repulsion among particles, thereby preventing aggregation. In general, nanocarriers with a zeta potential absolute value greater than 30 mV are considered physically stable [25]. The incorporation of OEO enhanced the negative charge of the liposomes to −39.8 mV, suggesting improved colloidal stability.

#### 3.1.2. FT-IR Spectroscopy

FT-IR spectroscopy was employed to analyze the encapsulation of OEO within the liposomes. The FT-IR spectra of Lip, OEO-Lip, OEO, PM, and their constituent materials are presented in Figure 1D. The spectrum of Lip exhibited characteristic peaks of egg yolk lecithin at 2925 cm^−1^, 2852 cm^−1^, 1736 cm^−1^, and 1243 cm^−1^. The peaks at 2925 cm^−1^ and 2852 cm^−1^ are attributed to the vibrational absorption of CH_2_ groups in the phospholipid hydrophobic tails [23]. The peak at 1243 cm^−1^ arises from asymmetric P=O stretching vibration, while the strong absorption at 1736 cm^−1^ corresponds to the C=O stretching vibration of the phospholipid carbonyl groups. Characteristic peaks of Tween 80 were observed at 2967 cm^−1^ and 1737 cm^−1^, and those of cholesterol were identified at 1376 cm^−1^ and 1065 cm^−1^. The presence of these peaks in the Lip spectrum confirms the successful formation of liposomes from lecithin, cholesterol, and Tween 80 [26]. The spectrum of pure OEO showed its characteristic absorption bands, including C-C skeletal vibrations of the aromatic ring (1502–1621 cm^−1^), –OCH_2_ stretching vibration of flavonoid compounds (2959 cm^−1^), C–OH stretching vibration of phenolics (3371 cm^−1^), ester group vibration of hydrocarbons (1726 cm^−1^), and ether vibration (1252 cm^−1^). Critical evidence for successful encapsulation was observed in the spectrum of OEO-Lip, where characteristic peaks of OEO were present. The overall spectral profile of OEO-Lip closely resembled that of blank Lip, with differences primarily noted as slight shifts in the positions of specific bands. This indicates that the encapsulation of OEO did not alter the fundamental structure of the liposomal matrix, with the observed spectral differences likely resulting from molecular vibrations or bending induced during the encapsulation process [27]. Furthermore, a noticeable broadening of the absorption band at approximately 3385 cm^−1^ in the OEO-Lip spectrum suggests the formation of hydrogen bonds between OEO and the liposomal components. This interaction provides further evidence for the successful encapsulation of OEO within the liposomes.

#### 3.1.3. XRD Analysis

XRD analysis was performed to investigate the crystallinity of Lip, OEO, OEO-Lip, and the PM. As shown in Figure 1E, the XRD pattern of OEO exhibited no distinct diffraction peaks, indicating its amorphous nature. In contrast, the PM sample displayed several sharp diffraction peaks within the 2θ range of 0–30°, along with a broad amorphous halo, suggesting a semi-crystalline structure. Both Lip and OEO-Lip showed sharp diffraction peaks at 2θ values of approximately 7°, 27°, 31°, 45°, 53°, and 56°, indicating a polymorphic crystalline structure. These peaks are attributed to the ordered lipid bilayer arrangement in the liposomes [28]. The similarity in the diffraction patterns of OEO-Lip and Lip suggests that the incorporation of OEO did not alter the crystalline behavior of the liposomal matrix. Moreover, the absence of characteristic OEO diffraction peaks in the OEO-Lip sample confirms that OEO remains in an amorphous state within the liposomes. Notably, the original diffraction peaks observed in the PM sample were no longer present in the Lip and OEO-Lip patterns. Instead, the liposomal formulations exhibited diffraction peaks at higher 2θ angles, indicating a more compact and ordered structure. The sharp, high-intensity peaks and stable baseline further suggest a high degree of crystallinity and structural regularity [29]. The encapsulation of OEO within a highly ordered crystalline lipid matrix is beneficial for enhancing its delivery performance [30] and contributes to the improved stability of OEO-Lip from a molecular structural perspective.

#### 3.1.4. Thermal Properties

DSC was employed to analyze the heat flow changes associated with the thermal behavior of liposomes upon loading with OEO. Liposomes can exhibit various liquid crystalline phases, and their phase transition temperature reflects the fluidity of the phospholipid bilayer [31]. The main phase transition temperature corresponds to the transformation of the liposomal membrane from the gel phase to the liquid crystalline phase. As illustrated in Figure 1F, the main phase transition temperature of OEO-Lip was 140.06 °C, which is approximately 18 °C lower than that of blank liposomes (Lip, 158.28 °C). A decrease in the phase transition enthalpy was also observed, which indicates the incorporation of hydrophobic compounds into the hydrophobic region of the phospholipid bilayer reduces both the phase transition temperature and enthalpy [32]. This phenomenon can be attributed to a decrease in intermolecular van der Waals forces due to hydrogen bonding between OEO and the polar head groups of phospholipids. Furthermore, no characteristic endothermic peak of pure OEO was detected at 175.65 °C in the OEO-Lip thermogram. Instead, a new endothermic peak appeared at 188.34 °C, suggesting that the incorporation of OEO into liposomes involves specific interactions rather than simple physical mixing.

TGA and derivative thermogravimetry (DTG) profiles of Lip, OEO, OEO-Lip, and PM are presented in Figure 1G. OEO exhibited a single mass-loss stage, with over 99% of its mass lost between 25 °C and 168.7 °C, indicating its volatile nature and low thermal stability under elevated temperatures. In contrast, PM, Lip, and OEO-Lip each displayed three distinct mass-loss stages. The initial stage was attributed to the evaporation of residual moisture. The second stage corresponded to the progressive thermal degradation of liposomal components. The third stage involved further decomposition through processes such as desulfurization, deacetylation, and depolymerization, ultimately leading to char formation [33]. The TGA and DTG curves of OEO-Lip closely resembled those of blank Lip, suggesting that the encapsulation of OEO did not significantly alter the thermal behavior of the liposomal matrix. Notably, OEO-Lip exhibited a markedly lower mass-loss rate compared to PM, reflecting its superior thermal stability. This enhancement is likely due to the formation of hydrogen bonds among egg yolk lecithin, cholesterol, and OEO within the liposomal structure, which improves the overall stability of the system. These findings are consistent with the interactions previously observed in the FT-IR analysis.

### 3.2. Storage Stability of OEO-Lip

#### 3.2.1. Morphology

**Figure 2:**
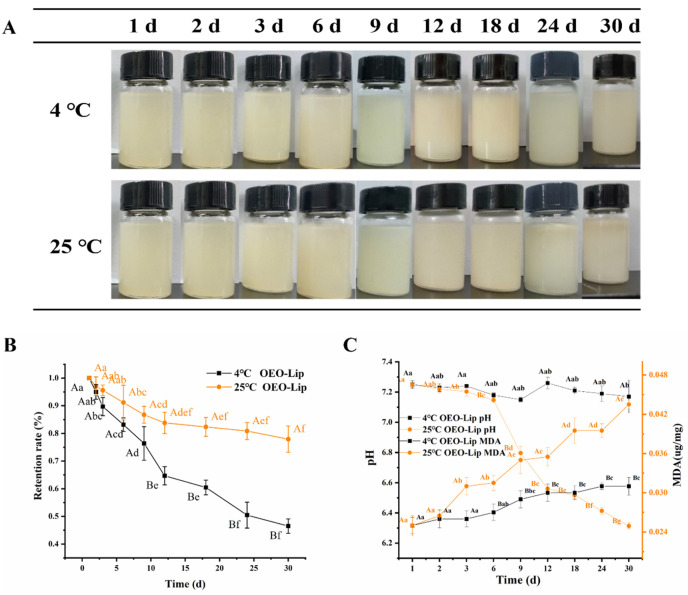
Storage stability of OEO-Lip. (**A**) Variation in the appearance of OEO-Lip at 4 °C and 25 °C over 30 d. (**B**) Retention of OEO-Lip at 4 °C and 25 °C over 30 d. (**C**) pH and MDA of OEO-Lip at 4 °C and 25 °C over 30 d. Different lowercase letters indicate significant differences among different days, and different uppercase letters indicate significant differences among different samples (*p* < 0.05).

Storage stability observations revealed a marked difference between the two temperatures (Figure 2A). The appearance of OEO-Lip stored at 4 °C remained largely unchanged throughout the 30-day study. Conversely, storage at 25 °C led to evident turbidity after 9 days, with further deterioration marked by phase separation, stratification, and precipitation after 24 days.

#### 3.2.2. Particle Size, Zeta Potential, PDI, and EE

The stability of OEO-Lip was evaluated by monitoring changes in particle size, zeta potential, PDI, and EE over 30 days of storage at 4 °C and 25 °C (Table 1). The results indicated OEO-Lip stored at 4 °C maintained relatively stable physicochemical properties throughout the storage period. In contrast, samples stored at 25 °C exhibited a considerable increase in average particle size, absolute zeta potential value, and PDI, along with a significant decrease in EE (*p* < 0.05). The zeta potential of OEO-Lip changed from –34.45 mV to –33.13 mV at 4 °C, and to –64.93 mV at 25 °C. Although the absolute zeta potential remained above 30 mV under both conditions, its fluctuation was minimal (within 5 mV) at 4 °C. In contrast, the pronounced increase in negative charge at 25 °C was accompanied by visible phase separation and flocculation. This suggests increased exposure of phospholipids and leakage of negatively charged OEO during storage may have contributed to the elevated surface charge and instability [34]. The average particle size increased from 186.60 nm to 221.72 nm at 4 °C, and to 2490.67 nm at 25 °C. The PDI rose from 0.22 to 0.25 at 4 °C, and reached 1.0 at 25 °C. The growth in particle size and PDI is likely due to the fusion and aggregation of liposomal membranes during storage. At 4 °C, the minimal change in PDI (remaining below 0.3) suggests that the liposomes effectively resisted aggregation under refrigerated conditions. In contrast, the PDI of 1.0 at 25 °C reflects a completely heterogeneous size distribution. The encapsulation efficiency of OEO-Lip decreased from 77.52% to 60.61% at 4 °C—a loss of only about 16%—whereas at 25 °C, it dropped markedly to 32.97%. The greater leakage at elevated temperature can be attributed to accelerated molecular motion of the lipid membrane and increased fluidity of the bilayer, facilitating the diffusion of encapsulated OEO [35].

#### 3.2.3. Retention Rate

The retention rate of OEO in OEO-Lip was measured over a 30-day storage period at 4 °C and 25 °C, as shown in Figure 2B. The results demonstrate that the OEO retention rate decreased gradually over time under both conditions, indicating that liposomal encapsulation can effectively protect the active components of the essential oil and reduce its loss during storage. The decline in OEO retention was significantly slower at 4 °C than at 25 °C. After 30 days, the retention rate remained as high as 77.93% at 4 °C, which was significantly greater than that at 25 °C (46.5%; *p* < 0.05). The higher retention observed at refrigerated temperature may be attributed to reduced membrane permeability, lower molecular mobility, and suppressed oxidative degradation of unsaturated fatty acids in the phospholipid bilayer [36]. These findings suggest that OEO-Lip is more suitable for storage under refrigerated conditions.

#### 3.2.4. pH and MDA Content

The stability of liposomal formulations during storage is primarily compromised by the degradation of phospholipids in the lipid bilayer, mainly through oxidation and hydrolysis [37]. Unsaturated fatty acids in phospholipids are susceptible to oxidation via free radical reactions, accelerated by temperature, light, and oxygen. Meanwhile, hydrolysis of ester bonds in phospholipids generates free fatty acids, which can lower the pH of the system [38]. Therefore, monitoring changes in pH can serve as an effective indicator of the physicochemical stability of OEO-Lip during storage. As shown in Figure 2C, the initial pH of the OEO-Lip system was near neutral (approximately 7.2). Under storage at 4 °C, the pH decreased only slightly from 7.29 to 7.14 over 30 days, indicating good stability. In contrast, at 25 °C, the pH declined noticeably over time. While little change was observed within the first 6 days, a clear divergence between storage conditions emerged by day 9, with the pH dropping to a minimum of 6.32 by day 30. This pronounced decrease coincided with observed changes in morphology, particle size, zeta potential, PDI, and retention rate, suggesting a correlation between pH reduction and overall physical instability. MDA, an intermediate product of lipid oxidation, was used as a key indicator of the extent of phospholipid degradation. The MDA content in OEO-Lip increased gradually during storage. At 4 °C, the rise was minimal, from 0.025 mg/g to 0.031 mg/g, which was significantly lower (*p* < 0.05) than the increase observed in samples stored at 25 °C. The MDA content exhibited an initial increase followed by a plateau in later stages. This trend can be explained by the fact that lipid oxidation is a chain reaction, and MDA is an intermediate; as oxygen in the system is depleted, the oxidation rate slows, leading to stabilization of MDA levels [39]. OEO-Lip stored at 4 °C exhibited only minor changes in particle size, Zeta potential, PDI, retention rate, visual appearance, and bilayer integrity over 30 days, confirming that the formulation possesses significantly greater stability under refrigerated conditions.

### 3.3. Antioxidant Activity of OEO-Lip

**Figure 3:**
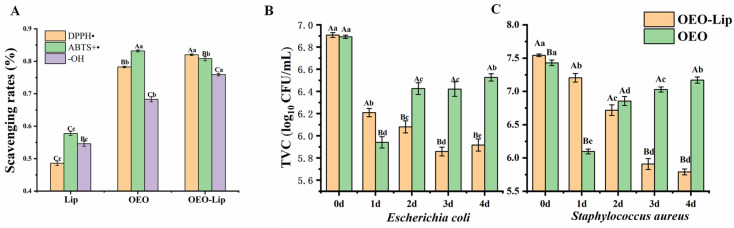
Antioxidant and antimicrobial activity of OEO-Lip. (**A**) Scavenging rates DPPH, ABTS^+^ and OH free radicals. Inhibitory effects on (**B**) *Escherichia coli* and (**C**) *Staphylococcus aureus* of OEO-Lip treatment over 4 d. Different lowercase letters indicate significant differences among different days, and different uppercase letters indicate significant differences among different samples (*p* < 0.05).

Both OEO-Lip and free OEO demonstrated excellent antioxidant activity in free radical scavenging assays, which can be attributed to active components in OEO—such as phenolic hydroxyl groups—that scavenge free radicals, chelate metal ions, quench reactive oxygen species, and interrupt free radical chain reactions [40]. During the antioxidant assays, when the free radical solution was introduced to the liposomal dispersion, the solvent molecules permeated the lipid bilayer, causing vesicle swelling and promoting the release of OEO, which subsequently neutralized the free radicals (Figure 3A). In the ABTS^+^ scavenging assay, free OEO showed a slightly higher activity (83.21%) than OEO-Lip (80.84%). In contrast, OEO-Lip exhibited stronger DPPH scavenging activity (82.03%) compared to free OEO (78.28%), and also outperformed free OEO in hydroxyl radical (•OH) scavenging. These differences may be attributed to variations in the extent to which the liposomal phospholipid bilayer interferes with different types of free radicals. Importantly, the results confirm that encapsulation within liposomes does not significantly compromise the antioxidant activity of OEO.

### 3.4. Antimicrobial Activity and Mechanism of OEO-Lip

Figure 3B,C illustrate the inhibitory effects of OEO and OEO-Lip on the growth of *E. coli* and *S. aureus* over a 4-day period. OEO initially reduced bacterial concentrations to 5.94 log CFU/mL (*E. coli*) and 6.10 log CFU/mL (*S. aureus*) on day 1; however, regrowth occurred subsequently, reaching 6.53 log CFU/mL and 7.17 log CFU/mL by day 4. This transient suppression may be attributed to the antimicrobial activity of key components in OEO such as carvacrol and thymol, whose efficacy diminishes over time due to volatility and instability [8]. In contrast, OEO-Lip demonstrated sustained and enhanced antibacterial performance. Although its initial inhibition on day 1 was slightly weaker than that of OEO, bacterial concentrations decreased continuously throughout the experiment, declining to 5.92 log CFU/mL (*E. coli*) and 5.79 log CFU/mL (*S. aureus*) by day 4, respectively. These results indicate that liposomal encapsulation provides controlled release of OEO, prolonging its antimicrobial activity. Furthermore, OEO-Lip exhibited stronger inhibition against *S. aureus* than against *E. coli*, likely due to the less complex cell wall structure of Gram-positive bacteria, which facilitates penetration of the active compounds.

#### 3.4.1. Microbial Growth Curves and Bacterial Morphology

**Figure 4:**
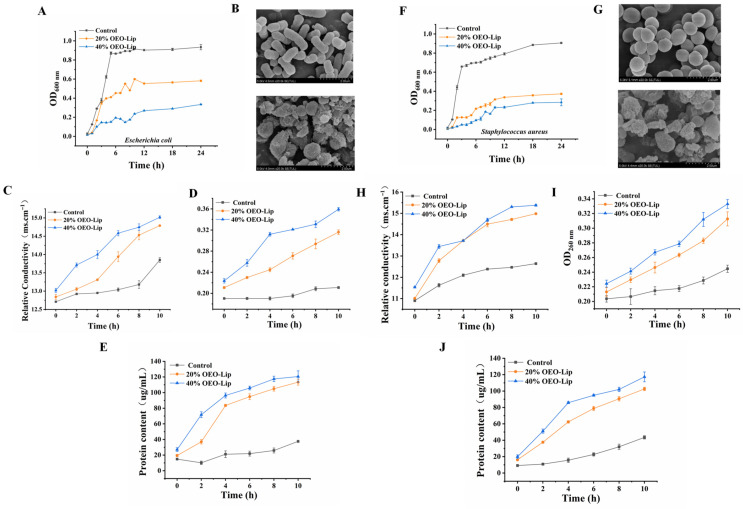
Antimicrobial mechanism of OEO-Lip. Growth curves of (**A**) *Escherichia coli* and (**F**) *Staphylococcus aureus* after exposure to OEO-Lip over 24 h. SEM of (**B**) *Escherichia coli* and (**G**) *Staphylococcus aureus* after 24 h of OEO-Lip treatment. Changes in relative conductivity induced by OEO-Lip treatment of (**C**) *Escherichia coli* and (**H**) *Staphylococcus aureus* over 10 h. RNA leakage from (**D**) *Escherichia coli* and (**I**) *Staphylococcus aureus* after OEO-Lip treatment over 10 h. Protein leakage from (**E**) *Escherichia coli* and (**J**) *Staphylococcus aureus* after OEO-Lip treatment over 10 h.

Analysis of the growth curves (Figure 4A,F) demonstrated that while free OEO exhibited initial antibacterial activity—reducing bacterial concentrations to 5.94 log CFU/mL (*E. coli*) and 6.10 log CFU/mL (*S. aureus*) on day 1—its efficacy diminished over time. By day 4, bacterial counts rebounded to 6.53 log CFU/mL and 7.17 log CFU/mL, respectively, indicating a time-dependent decline in efficacy, likely due to volatility and inactivation of active components. In contrast, OEO-Lip displayed sustained antibacterial performance, with bacterial concentrations progressively decreasing to 5.92 log CFU/mL (*E. coli*) and 5.79 log CFU/mL (*S. aureus*) by day 4. This suggests that liposomal encapsulation enables controlled release of active compounds, prolonging antimicrobial action. TEM observations further revealed structural damage to bacteria following OEO-Lip treatment. *E. coli* lost their rod-shaped morphology, showing disrupted cellular structures and rough vesicle-like forms (Figure 4B), while most *S. aureus* cells appeared shriveled, ruptured, or lysed (Figure 4G). These results indicate that the antimicrobial mechanism of OEO involves disruption of bacterial structural integrity, leading to bacterial death.

#### 3.4.2. Bacterial Cell Membrane Permeability

Intracellular electrolytes play crucial roles in regulating cellular energy metabolism and solute transport, making the maintenance of ionic homeostasis essential for proper cell function. The cell membrane acts as a permeability barrier for ions such as Na^+^, K^+^, and Ca^2+^. When antibacterial agents act on bacterial cells, membrane integrity is compromised, leading to the leakage of electrolytes and a consequent increase in extracellular conductivity [41]. Thus, conductivity serves as an important indicator of cellular integrity and membrane permeability. As shown in Figure 4C,H, the conductivity of bacterial suspensions treated with OEO-Lip increased rapidly and was significantly higher than that of the blank control group (*p* < 0.05). These results demonstrate that OEO-Lip treatment damages the cell membranes of both *S. aureus* and *E. coli*, resulting in ion leakage and elevated conductivity.

#### 3.4.3. The Leakage of RNA and Proteins

Exposure to OEO-Lip resulted in the disruption of bacterial cell walls and membranes, leading to the leakage of intracellular components, including nucleic acids and proteins. As depicted in Figure 4D,I, the extracellular RNA content of both *S. aureus* and *E. coli* increased rapidly after OEO-Lip treatment. Furthermore, the extent of cellular leakage exhibited a concentration-dependent relationship with the dosage of OEO-Lip. After 10 h of treatment, the extracellular protein content of *S. aureus* rose from an initial 9.14 μg/mL to 43.48 μg/mL (Figure 4E), while that of *E. coli* increased from 14.86 μg/mL to 37.56 μg/mL (Figure 4J). These values were significantly higher than those observed in untreated controls (*p* < 0.05). These findings indicate that OEO-Lip treatment compromises bacterial membrane integrity, causing irreversible damage and subsequent leakage of intracellular substances. Since the leakage of proteins and RNA requires irreversible lysis of the cell membrane, and as these macromolecules cannot traverse the membrane via normal transport channels, their release into the culture medium occurs only when the membrane is severely disrupted or structurally compromised [42]. In the experiment, treatment with 20% OEO-Lip only increased membrane permeability without reaching the level of membrane rupture; thus, the leakage of proteins and RNA in this group remained almost identical to that of the control. In contrast, 40% OEO-Lip significantly intensified membrane damage, leading to the rupture of the bacterial membrane and consequent massive leakage of intracellular proteins and RNA, which resulted in a marked increase in leakage levels.

#### 3.4.4. Transcriptomic Analysis of the Antimicrobial Mechanism

**Figure 5:**
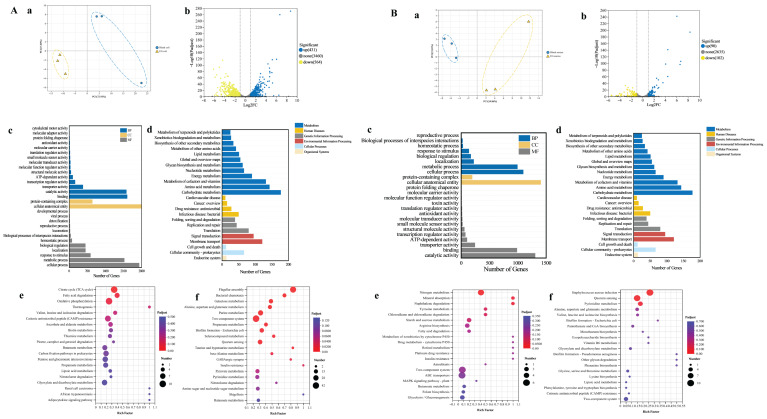
Transcriptomic analysis of (**A**) *Escherichia coli* and (**B**) *Staphylococcus aureus* in response to OEO-Lip treatment. (**a**) PCA score plot; (**b**) Statistics of differentially expressed genes (padj < 0.05); (**c**) GO enrichment analysis of DEGs; (**d**) KEGG pathway analysis of DEGs; (**e**) KEGG enrichment for up-regulated genes; (**f**) KEGG enrichment for down-regulated genes.

To elucidate the mechanism by which OEO-Lip disrupts bacterial cell membranes, RNA-Seq was performed to analyze its impact on gene expression in *E. coli* and *S. aureus*. Principal component analysis (PCA) revealed distinct transcriptomic profiles between OEO-Lip-treated and control groups for both bacteria (Figure 5A(a),B(a)). Volcano plots identified 995 differentially expressed genes (DEGs) in *E. coli* (431 up- and 564 down-regulated; Figure 5A(b)) and 192 DEGs in *S. aureus* (90 up- and 102 down-regulated; Figure 5B(b)). Gene Ontology (GO) enrichment analysis indicated that in *E. coli*, DEGs were primarily associated with biological processes such as cellular and metabolic processes, localized to cellular anatomical entities, and involved in binding functions (Figure 5A(c)). In *S. aureus*, DEGs were mainly enriched in cellular processes, cellular anatomical entities, and catalytic activity (Figure 5B(c)). Kyoto Encyclopedia of Genes and Genomes (KEGG) pathway analysis further demonstrated that up-regulated DEGs in *E. coli* were enriched in the citrate cycle (TCA cycle), fatty acid degradation, and oxidative phosphorylation, while down-regulated DEGs were significantly associated with flagellar assembly and two-component systems (Figure 5A(e,f)). In *S. aureus*, up-regulated genes were related to nitrogen metabolism, whereas down-regulated genes were enriched in quorum sensing (Figure 5B(e,f)). These findings suggest that OEO-Lip exerts antibacterial effects through coordinated interference with bacterial motility, substance transport, and energy metabolism.

Further analysis revealed that OEO-Lip significantly altered the expression of membrane-related genes in *E. coli* (Table 2). Key genes involved in phospholipid synthesis (e.g., *gpsA*, *pgpC*, *glpA*, *eutA/B/C*) were down-regulated, inhibiting phospholipid synthesis and ethanolamine metabolism, thereby compromising membrane integrity [43]. Concurrently, genes within the fad regulon were up-regulated, promoting fatty acid degradation [44]. Membrane transport systems were also affected: 33 DEGs were enriched in ABC transporter pathways and 8 in phosphotransferase system (PTS) pathways. Genes encoding transporters for sugars, nickel ions, and oligopeptides (e.g., *potD, rbsB, nikA-E*) were generally down-regulated, restricting nutrient uptake [45]. In *S. aureus* (Table 3), OEO-Lip disrupted multiple membrane transport functions. Down-regulation of genes such as *metN*, *oppB*, *gatC*, and *ecfT* impaired the transport of methionine, oligopeptides, sugars, and biotin, inhibiting bacterial growth [46,47]. Conversely, up-regulation of efflux pump genes (e.g., *bceA*, *hrtA/B*) [48] and membrane permeability-related genes (*opuA*) [49] may exacerbate intracellular leakage and induce stress responses. OEO-Lip impairs bacterial cell membrane function through multiple pathways, including suppression of membrane lipid synthesis, disruption of nutrient transport, and increased membrane permeability, collectively leading to growth inhibition and cell death.

### 3.5. Sustained-Release Properties in Food Simulants of OEO-Lip

**Figure 6:**
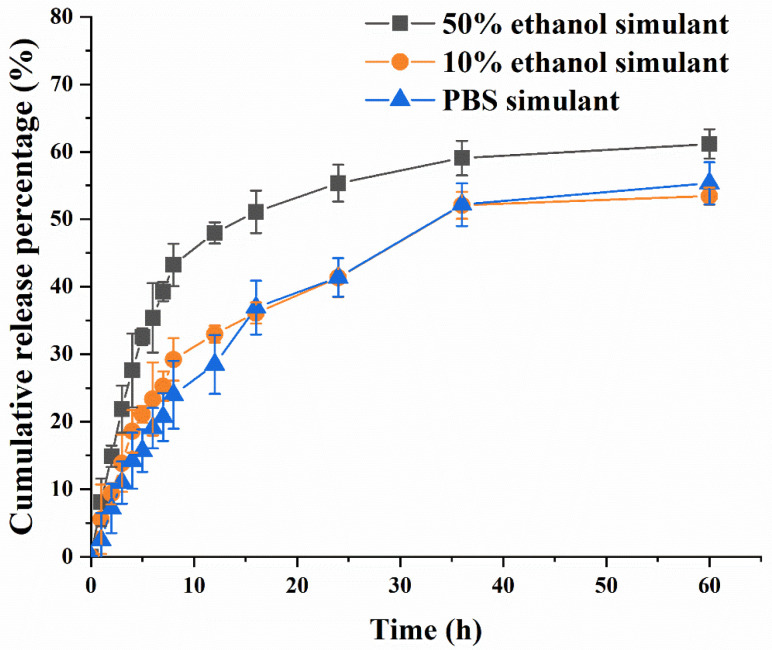
Cumulative release percentage of OEO-Lip in 50% ethanol, 10% ethanol and PBS.

The release kinetics of the prepared OEO-Lip were evaluated in different food simulants. In this study, 10% ethanol was selected to simulate hydrophilic foods, 50% ethanol was used to represent alcoholic beverages (with >20% alcohol content) and oil-in-water emulsions, and PBS served as a simulant for neutral-pH aqueous foods. The release study was conducted according to the European Union regulation (EU) No 10/2011. As shown in Figure 6, the cumulative release rate of OEO increased gradually over time in all three release media and began to stabilize after 15 h. After 36 h. A high initial release rate was observed within the first 6 h in all media, which can be attributed to the rapid release of OEO associated with the liposome surface—a characteristic “burst release” phenomenon. In the prepared OEO-Lip, OEO is encapsulated in two distinct forms: surface-adsorbed and internally entrapped. The surface-adsorbed OEO, attached to the liposome membrane via hydrophobic interactions and van der Waals forces, exhibits weak binding affinity and rapidly diffuses into the release medium, corresponding to the initial rapid rise in the release curve [50]. In contrast, the internally entrapped OEO is completely embedded within the hydrophobic core or the interlamellar spaces of the liposomal bilayer, where it forms stronger associations with phospholipid molecules. The release of this fraction requires overcoming both steric hindrance from the liposomal membrane and intermolecular interactions, making it difficult to fully diffuse into the medium under static release conditions [51]. Consequently, this portion remains largely retained, leading to a plateau in the overall release profile at approximately 60%. Compared to 10% ethanol and PBS, the release was relatively faster in 50% ethanol. This is likely due to the higher ethanol concentration enhancing permeability into the liposomal bilayer, thereby promoting the diffusion of encapsulated OEO [52]. The release medium used in this experiment was phosphate-buffered saline (PBS, an aqueous system), while OEO is a typical hydrophobic essential oil with poor compatibility with aqueous media [53]. During the release process, the hydrophobic OEO molecules dissociated from the liposomes tend to undergo micro-aggregation in the aqueous medium, hindering their sustained diffusion into the bulk medium. The release profiles demonstrate that the liposomal encapsulation effectively retained OEO, with the lipid bilayer acting as a barrier that delayed release and extended the duration of OEO delivery. The release data were fitted to four kinetic models: zero-order, first-order, Higuchi, and Ritger–Peppas (Table 4). The results indicated that the release of OEO-Lip in all three media best followed the first-order kinetic model, with R^2^ values exceeding 0.97, consistent with the typical release behavior of sustained-release formulations. From the perspective of release kinetics, OEO-Lip exhibits superior release characteristics in 50% ethanol, a simulant of alcoholic or emulsified food matrices. However, considering the physical stability of liposomes in hydrophilic systems and the goal of serving as a sustained-release carrier, their application in liquid or semi-solid hydrophilic foods with neutral pH may potentially extend the duration of antimicrobial activity. Nevertheless, attention should be paid to the potential limitation in the effective release amount of OEO.

## 4. Conclusions

This study successfully developed and comprehensively evaluated a liposomal delivery system (OEO-Lip) for the encapsulation of oregano essential oil. Comprehensive characterization confirmed the successful incorporation of OEO into the liposomes, resulting in the formation of structurally stable and uniformly dispersed nanoparticles. Compared to free OEO, OEO-Lip exhibited significantly enhanced physical and thermal stability, maintaining stable physicochemical properties over extended periods, particularly under refrigerated storage at 4 °C. Liposomal encapsulation not only preserved the inherent antioxidant capacity of OEO but also conferred stronger and more prolonged broad-spectrum antimicrobial activity. The enhanced efficacy is achieved through disruption of bacterial cell membrane integrity, induction of intracellular content leakage, and transcriptomic-level modulation of key pathways, including phospholipid synthesis, nutrient transport, quorum sensing, and energy metabolism. Furthermore, OEO-Lip demonstrated ideal sustained-release properties in food simulants. Consequently, this study provides an efficient delivery strategy for OEO, indicating that OEO-Lip holds considerable promise as a natural preservative for application in the field of food preservation.

## Figures and Tables

**Table 1:** Changes in particle size, zeta potential, PDI, and embedding efficiency (EE) of OEO-Lip at different temperatures.

	Time (d)	Particle Size (nm)	Zeta Potential (mV)	PDI	EE (%)
4 °C	1	186.60 ± 12.86 ^Aa^	−34.45 ± 3.10 ^Aa^	0.22 ± 0.06 ^Aa^	77.52 ± 1.30 ^Aa^
	2	203.80 ± 13.14 ^Aa^	−36.10 ± 3.70 ^Aa^	0.21 ± 0.02 ^Aa^	75.59 ± 3.11 ^Aab^
	3	191.90 ± 11.34 ^Aa^	−34.00 ± 13.20 ^Aa^	0.19 ± 0.01 ^Aa^	74.47 ± 1.52 ^Aab^
	6	196.70 ± 25.18 ^Aa^	−35.15 ± 6.10 ^Aa^	0.20 ± 0.01 ^Aa^	71.22 ± 0.80 ^Abc^
	9	213.30 ± 59.12 ^Ba^	−34.11 ± 1.70 ^Aa^	0.24 ± 0.03 ^Ba^	67.33 ± 2.60 ^Acd^
	12	210.66 ± 21.45 ^Ba^	−30.62 ± 7.30 ^Aa^	0.21 ± 0.06 ^Ba^	65.20 ± 1.89 ^Ade^
	18	211.55 ± 13.45 ^Ba^	−33.65 ± 4.90 ^Aa^	0.21 ± 0.02 ^Ba^	63.95 ± 0.43 ^Ade^
	24	215.20 ± 17.26 ^Ba^	−35.02 ± 3.90 ^Aa^	0.21 ± 0.04 ^Ba^	62.98 ± 3.88 ^Ade^
	30	221.72 ± 43.65 ^Ba^	−33.13 ± 7.10 ^Aa^	0.25 ± 0.03 ^Ba^	60.61 ± 2.30 ^Ae^
25 °C	1	186.60 ± 12.86 ^Af^	−34.45 ± 3.10 ^Aa^	0.23 ± 0.03 ^Ad^	77.52 ± 1.31 ^Aa^
	2	224.25 ± 19.26 ^Af^	−36.08 ± 2.70 ^Aa^	0.22 ± 0.02 ^Ad^	69.68 ± 1.47 ^Ab^
	3	205.87 ± 9.21 ^Af^	−34.17 ± 0.90 ^Aa^	0.20 ± 0.01 ^Ad^	62.05 ± 3.67 ^Bb^
	6	213.47 ± 25.13 ^Af^	−36.33 ± 2.10 ^Aa^	0.18 ± 0.07 ^Ad^	59.57 ± 1.11 ^Bc^
	9	578.88 ± 7.15 ^Ae^	−49.48 ± 3.10 ^Bb^	0.50 ± 0.03 ^Ac^	50.47 ± 3.49 ^Bc^
	12	1030.00 ± 31.54 ^Bd^	−45.50 ± 4.30 ^Bb^	0.64 ± 0.05 ^Ab^	46.88 ± 2.24 ^Bd^
	18	1785.40 ± 50.78 ^Bc^	−60.44 ± 5.50 ^Bc^	0.71 ± 0.07 ^Ab^	46.94 ± 1.15 ^Bd^
	24	2156.00 ± 101.90 ^Bb^	−60.67 ± 3.90 ^Bc^	0.93 ± 0.12 ^Aa^	40.10 ± 0.93 ^Bd^
	30	2490.67 ± 353.40 ^Ba^	−64.93 ± 7.20 ^Bc^	0.92 ± 0.20 ^Aa^	32.97 ± 2.32 ^Be^

Note: Lowercase letters indicate significant differences over time within the same treatment, while uppercase letters indicate significant differences among different temperature treatments (*p* < 0.05).

**Table 2:** Differentially expressed genes related to cell membrane structure and function after OEO-Lip treatment of *Escherichia coli*.

Gene ID	Gene Name	Fold Change	Functional Description
Cellular Membrane Composition			
b3608	*gpsA*	0.43	glycerol-3-phosphate dehydrogenase
b2241	*glpA*	0.47	anaerobic glycerol-3-phosphate dehydrogenase subunit A
b2560	*pgpC*	0.37	phosphatidylglycerophosphatase C
b2441	*eutB*	0.12	ethanolamine ammonia-lyase subunit alpha
b2440	*eutC*	0.11	ethanolamine ammonia-lyase subunit beta
b2451	*eutA*	0.12	ethanolamine ammonia-lyase reactivase EutA
b1805	*fadD*	2.35	long-chain-fatty-acid--CoA ligase
b0221	*fadE*	5.33	acyl-CoA dehydrogenase
b3846	*fadB*	3.93	multifunctional enoyl-CoA hydratase 3-hydroxyacyl-CoA
b3845	*fadA*	5.32	3-ketoacyl-CoA thiolase
Cellular Transport			
b1123	*potD*	0.46	spermidine preferential ABC transporter periplasmic binding protein
b3751	*rbsB*	0.44	ribose ABC transporter periplasmic binding protein
b1901	*araF*	0.33	arabinose ABC transporter periplasmic binding protein
b1900	*araG*	0.47	arabinose ABC transporter ATP-binding subunit
b3476	*nikA*	0.23	Ni(2(+)) ABC transporter periplasmic binding protein
b3477	*nikB*	0.36	Ni(2(+)) ABC transporter membrane subunit NikB
b3478	*nikC*	0.06	Ni(2(+)) ABC transporter membrane subunit NikC
b3479	*nikD*	0.30	Ni(2(+)) ABC transporter membrane subunit NikD
b3480	*nikE*	0.39	Ni(2(+)) ABC transporter ATP binding subunit NikE
b1329	*oppA*	0.39	murein tripeptide ABC transporter periplasmic binding protein
b3544	*dppA*	0.47	dipeptide ABC transporter periplasmic binding protein
b3195	*mlaF*	2.21	intermembrane phospholipid transport system ATP-binding subunit MlaF
b3193	*mlaD*	2.02	intermembrane phospholipid transport system substrate binding protein MlaD
b3728	*pstS*	6.75	phosphate ABC transporter periplasmic binding protein
b3727	*pstC*	3.12	phosphate ABC transporter membrane subunit PstC
b3726	*pstA*	2.29	phosphate ABC transporter membrane subunit PstA
b3133	*agaV*	0.07	N-acetyl-D-galactosamine-specific PTS enzyme IIB component
b1818	*manY*	0.40	mannose-specific PTS enzyme IIC component

**Table 3:** Differentially expressed genes related to cell membrane structure and function after OEO-Lip treatment of *Staphylococcus aureus*.

Gene ID	Gene Name	Fold Change	Functional Description
Cellular Membrane Composition			
SAOUHSC_03006	*liP*	0.29	lipase
SAOUHSC_01710	*accB*	0.31	acetyl-CoA carboxylase biotin carboxyl carrier protein subunit
SAOUHSC_00608	*adhP*	2.59	alcohol dehydrogenase
SAOUHSC_00113	*adhE*	3.18	bifunctional acetaldehyde-CoA/alcohol dehydrogenase
Cellular Transport			
SAOUHSC_00923	*oppB*	0.40	Oligopetide transporter-associated protein
SAOUHSC_03018	*ecfT*	0.41	Biotin transporter-associated protein
SAOUHSC_00423	*metN*	0.41	D-Methionine transporter-associated protein
SAOUHSC_00216	*gatC*	0.44	PTS system transporter
SAOUHSC_00556	*aid39071.1*	2.00	proline/betaine transporter
SAOUHSC_02744	*opuA*	3.07	amino acid ABC transporter ATP-binding protein
SAOUHSC_03036	*bceA*	4.17	ABC transporter ATP-binding protein
SAOUHSC_02641	*hrtB*	16.66	permease domain-containing protein
SAOUHSC_02640	*hrtA*	21.678	hypothetical protein

**Table 4:** Fitting results of OEO-Lip in sustained-release kinetic models in different simulated releases.

Kinetic Model	10% Ethanol Simulant		50% Ethanol Simulant		PBS Simulant	
	Fitted Equation	R^2^	Fitted Equation	R^2^	Fitted Equation	R^2^
Zero-order	Mt = 0.8422t + 14.7976	0.756	Mt = 0.8716t + 24.0729	0.582	Mt = 0.9404t + 11.1047	0.820
First-order	Mt = 51.152(1 − e^−0.09517t^)	0.978	Mt = 58.555(1 − e^−0.15489^)	0.995	Mt = 56.061(1 − e^−0.0652t^)	0.994
Higuchi equation	Mt = 7.6353t^1/2^ + 2.7344	0.938	Mt = 8.4743t^1/2^ + 9.8536	0.822	Mt = 8.2782t^1/2^ − 1.6160	0.961
Ritger–Peppas model	Mt = 10.7195t^0.4173^	0.956	Mt = 18.6565t^0.3226^	0.901	Mt = 7.7532t^0.5061^	0.958

Note: R^2^ denotes the coefficient of determination.

## Data Availability

The original contributions presented in the study are included in the article/Supplementary Materials. Further inquiries can be directed to the corresponding authors.

## Supplementary Materials

The following supporting information can be downloaded at https://www.mdpi.com/article/10.3390/foods15010157/s1. Figure S1: Results of single-factor experiment. (A) Lecithin to cholesterol mass ratio. (B) OEO concentration. (C) Tween 80 concentration. (D) pH of PBS solution. (E) Volume of PBS buffer. (F) Rotary evaporation temperature. a–c indicated the results of one-way ANOVA for comparisons; Figure S2: Influence of interaction among factors on embedding rate. (A) A: OEO concentration and B: PBS volume; (B) B: PBS volume and C: Rotary evaporation temperature; (C) A: OEO concentration and C: Rotary evaporation temperature; (D) B: PBS volume and D: Lecithin to cholesterol mass ratio; (E) A: OEO concentration and D: Lecithin to cholesterol mass ratio; (F) C: Rotary evaporation temperature and D: Lecithin to cholesterol mass ratio. Table S1-1: Response surface test factors and levels; Table S1-2: Response surface test factors and levels; Table S1-3: Response surface optimization test results; Table S1-4: Response surface analysis of variance.

## References

[1] European Food Safety Authority, European Centre for Disease Prevention and Control (2023). The European Union One Health 2022 Zoonoses Report. EFSA J..

[2] Pakbin B., Brück W.M., Rossen J.W.A. (2021). Virulence Factors of Enteric Pathogenic *Escherichia coli*: A Review. Int. J. Mol. Sci..

[3] Li Q., Dou L., Zhang Y., Luo L., Yang H., Wen K., Yu X., Shen J., Wang Z. (2024). A comprehensive review on the detection of Staphylococcus aureus enterotoxins in food samples. Compr. Rev. Food Sci. Food Saf..

[4] Qi J., Hu C., Li J., Li Y., Zhang Y., Liu J., Xiao Y., Zhang W., Wei D., Liu J. (2025). Vancomycin-bacterial imprinted polymer hybrid for viable Staphylococcus aureus highly efficient capture, photothermal inactivation, and sensitive detection. Food Chem..

[5] Dubois L., Vettiger A., Buss J.A., Bernhardt T.G. (2025). Using fluorescently labeled wheat germ agglutinin to track lipopolysaccharide transport to the outer membrane in *Escherichia coli*. mBio.

[6] Kaur M., Mozaheb N., Paiva T.O., Herent M.F., Goormaghtigh F., Paquot A., Terrasi R., Mignolet E., Décout J.L., Lorent J.H. (2024). Insight into the outer membrane asymmetry of P. aeruginosa and the role of MlaA in modulating the lipidic composition, mechanical, biophysical, and functional membrane properties of the cell envelope. Microbiol. Spectr..

[7] Zinno P., Guantario B., Lombardi G., Ranaldi G., Finamore A., Allegra S., Mammano M.M., Fascella G., Raffo A., Roselli M. (2023). Chemical Composition and Biological Activities of Essential Oils from Origanum vulgare Genotypes Belonging to the Carvacrol and Thymol Chemotypes. Plants.

[8] Marianelli C., Ferraiuolo S., Topini M., Narciso L. (2025). In Vitro Evaluation of the Antimicrobial Activity of Eighteen Essential Oils Against Gram-Positive and Gram-Negative Bacteria in Two Different Growth Media. Pathogens.

[9] Nikolic I., Aleksic Sabo V., Gavric D., Knezevic P. (2024). Anti-Staphylococcus aureus Activity of Volatile Phytochemicals and Their Combinations with Conventional Antibiotics Against Methicillin-Susceptible *S. aureus* (MSSA) and Methicillin-Resistant *S. aureus* (MRSA) Strains. Antibiotics.

[10] Mączka W., Twardawska M., Grabarczyk M., Wińska K. (2023). Carvacrol—A Natural Phenolic Compound with Antimicrobial Properties. Antibiotics.

[11] Yang L., Chen W., Zhang X., Xiao R., Ding X., Jing Z., Zhang H. (2026). Construction and application of controlled-release preservation pads based on Zein/sodium caseinate-stabilized oregano essential oil Pickering emulsions. Food Hydrocoll..

[12] Zhao S., Li Y., Liu Q., Xia X., Chen Q., Liu H., Kong B. (2024). Characterization, release profile, and antibacterial properties of oregano essential oil nanoemulsions stabilized by soy protein isolate/tea saponin nanoparticles. Food Hydrocoll..

[13] Anaya-Castro M.A., Ayala-Zavala J.F., Muñoz-Castellanos L., Hernández-Ochoa L., Peydecastaing J., Durrieu V. (2017). β-Cyclodextrin inclusion complexes containing clove (Eugenia caryophyllata) and Mexican oregano (Lippia berlandieri) essential oils: Preparation, physicochemical and antimicrobial characterization. Food Packag. Shelf Life.

[14] Yan D., Wang Y., Yang R., Man Y., Tang H., Yu Q., Shao L. (2025). Liposome-delivered natural antimicrobials in foods: Antibacterial efficacy and mechanisms. Food Biosci..

[15] Yang R., Zhang X., Si J., Zhao X., Song J. (2025). Liposome Technology in Food Science: Structure, Preparation Techniques, and Functional Applications. J. Future Foods.

[16] Ajeeshkumar K.K., Aneesh P.A., Raju N., Suseela M., Ravishankar C.N., Benjakul S. (2021). Advancements in liposome technology: Preparation techniques and applications in food, functional foods, and bioactive delivery: A review. Compr. Rev. Food Sci. Food Saf..

[17] Song F.-f., Tian S.-j., Yang G.-l., Sun X.-y. (2022). Effect of phospholipid/flaxseed oil ratio on characteristics, structure change, and storage stability of liposomes. LWT.

[18] Ben-Fadhel Y., Maherani B., Salmieri S., Lacroix M. (2022). Preparation and characterization of natural extracts-loaded food grade nanoliposomes. LWT.

[19] Huang R., Song H., Wang X., Shen H., Li S., Guan X. (2024). Fatty acids-modified liposomes for encapsulation of bioactive peptides: Fabrication, characterization, storage stability and in vitro release. Food Chem..

[20] Wang J., Jiang X., Feng L., Han J., Li Y., Wang X., Kitazawa H., Guo Y., Li L. (2025). Colloidal stability of Salvia officinalis essential oil nano-liposomes and its antioxidant and antibacterial dual effect in postharvest preservation of Agaricus bisporus. Food Control.

[21] Kumar A., Badgujar P.C., Mishra V., Sehrawat R., Babar O.A., Upadhyay A. (2019). Effect of microfluidization on cholesterol, thermal properties and in vitro and in vivo protein digestibility of milk. LWT.

[22] Verma K., Tarafdar A., Kumar D., Sari T.P., Badgujar P.C., Pareek S., Assadpour E., Jafari S.M. (2025). Microfluidization based nano/sub-micron curcumin formulations for food and nutraceuticals: Physico-functional characteristics and safety aspects. Food Chem..

[23] Li L.-W., Chen X.-Y., Liu L.-C., Yang Y., Wu Y.-J., Chen G., Zhang Z.-F., Luo P. (2021). Oil-in-water camellia seeds oil nanoemulsions via high pressure microfluidization: Formation and evaluation. LWT.

[24] Large D.E., Abdelmessih R.G., Fink E.A., Auguste D.T. (2021). Liposome composition in drug delivery design, synthesis, characterization, and clinical application. Adv. Drug Deliv. Rev..

[25] Apostolidis E., Gerogianni A., Anagnostaki E., Paximada P., Mandala I. (2024). Assembly of spherical-shaped resistant starch nanoparticles to the oil droplet surface promotes the formation of stable oil in water Pickering emulsions. Food Hydrocoll..

[26] Rocchio J., Neilsen J., Everett K., Bothun G.D. (2017). A solvent-free lecithin-Tween 80 system for oil dispersion. Colloids Surf. A Physicochem. Eng. Asp..

[27] Kayaci F., Uyar T. (2011). Solid Inclusion Complexes of Vanillin with Cyclodextrins: Their Formation, Characterization, and High-Temperature Stability. J. Agric. Food Chem..

[28] Qi Y., Wang C., Qian R., Chen M., Jiang P., Wang T., Wang N. (2021). Loading drugs into liposomes by temperature up-down cycle procedure with controllable results fitting prediction by mathematical and thermodynamic process. Mater. Sci. Eng. C.

[29] Li Z., Ge G., Yang J., Wang X., Li R., Xu L., Cheng Y., Hou L., Feng C., Meng J. (2024). Glucono-δ-lactone induced Auricularia auricula polysaccharide-casein composite gels for curcumin loading and delivery. Int. J. Biol. Macromol..

[30] Shashidhar G.M., Manohar B. (2018). Nanocharacterization of liposomes for the encapsulation of water soluble compounds from Cordyceps sinensis CS1197 by a supercritical gas anti-solvent technique. RSC Adv..

[31] Cheng L., Zhang M., Dong L., Wang Y., Dong J., Wu F., Zheng C., Ma Y., Wang Z., Wang R. (2025). Interaction between hyaluronic acid and phospholipid bilayer and its influence on stability and bioavailability of luteolin-loaded liposomes. Food Chem. X.

[32] Rashed M.M.A., Tong Q., Nagi A., Li J., Khan N.U., Chen L., Rotail A., Bakry A.M. (2017). Isolation of essential oil from Lavandula angustifolia by using ultrasonic-microwave assisted method preceded by enzymolysis treatment, and assessment of its biological activities. Ind. Crops Prod..

[33] Wang X., Huang J., Zhu X., Guo Q., Cui Q., Yu J., Hou X., Liu H., Shen G., He Z. (2026). Enhanced stability and antioxidant activity of pickering emulsions stabilized by chayote tuber starch-tea polyphenol composites. Food Hydrocoll..

[34] Keivani Nahr F., Ghanbarzadeh B., Hamishehkar H., Kafil H.S., Hoseini M., Moghadam B.E. (2019). Investigation of physicochemical properties of essential oil loaded nanoliposome for enrichment purposes. LWT.

[35] Lin L., Chen W., Li C., Cui H. (2019). Enhancing stability of Eucalyptus citriodora essential oil by solid nanoliposomes encapsulation. Ind. Crops Prod..

[36] Gibis M., Zeeb B., Weiss J. (2014). Formation, characterization, and stability of encapsulated hibiscus extract in multilayered liposomes. Food Hydrocoll..

[37] Frenzel M., Steffen-Heins A. (2015). Impact of quercetin and fish oil encapsulation on bilayer membrane and oxidation stability of liposomes. Food Chem..

[38] Duché G., Sanderson J.M. (2024). The Chemical Reactivity of Membrane Lipids. Chem. Rev..

[39] Huang J., Wang Q., Chu L., Xia Q. (2020). Liposome-chitosan hydrogel bead delivery system for the encapsulation of linseed oil and quercetin: Preparation and in vitro characterization studies. LWT.

[40] Wang H., Ren R., Li M., Gao Z., Li H., Li S. (2025). Coaxial emulsion electrospinning fiber films loaded with OEO for active packaging of pork. Food Res. Int..

[41] Lemoni Z., Evangeliou K., Lymperopoulou T., Mamma D. (2025). Incorporation of Edible Plant Extracts as Natural Food Preservatives: Green Extraction Methods, Antibacterial Mechanisms and Applications. Foods.

[42] Burt S. (2004). Essential oils: Their antibacterial properties and potential applications in foods—A review. Int. J. Food Microbiol..

[43] Song M., Chen S., Lin W., Zhu K. (2024). Targeting bacterial phospholipids and their synthesis pathways for antibiotic discovery. Prog. Lipid Res..

[44] Chu K.H., Huang G., An T., Li G., Yip P.L., Ng T.W., Yip H.Y., Zhao H., Wong P.K. (2016). Photocatalytic inactivation of *Escherichia coli*—The roles of genes in β-oxidation of fatty acid degradation. Catal. Today.

[45] Moussatova A., Kandt C., O’Mara M.L., Tieleman D.P. (2008). ATP-binding cassette transporters in *Escherichia coli*. Biochim. Biophys. Acta (BBA) Biomembr..

[46] Rossi F., Zotta T., Iacumin L., Reale A. (2016). Theoretical insight into the heat shock response (HSR) regulation in Lactobacillus casei and L. rhamnosus. J. Theor. Biol..

[47] Dayan O., Nagarajan A., Shah R., Ben-Yona A., Forrest L.R., Kanner B.I. (2017). An Extra Amino Acid Residue in Transmembrane Domain 10 of the γ-Aminobutyric Acid (GABA) Transporter GAT-1 Is Required for Efficient Ion-coupled Transport*. J. Biol. Chem..

[48] Wu H., Zhang Y., Li L., Li Y., Yuan L., E Y., Qiao J. (2022). Positive regulation of the DLT operon by TCSR7 enhances acid tolerance of Lactococcus lactis F44. J. Dairy Sci..

[49] Kempf B., Bremer E. (1995). OpuA, an Osmotically Regulated Binding Protein-dependent Transport System for the Osmoprotectant Glycine Betaine in Bacillus subtilis(∗). J. Biol. Chem..

[50] Hasheminya S.-M., Dehghannya J. (2021). Development and characterization of novel edible films based on Cordia dichotoma gum incorporated with Salvia mirzayanii essential oil nanoemulsion. Carbohydr. Polym..

[51] Hammoud Z., Gharib R., Fourmentin S., Elaissari A., Greige-Gerges H. (2019). New findings on the incorporation of essential oil components into liposomes composed of lipoid S100 and cholesterol. Int. J. Pharm..

[52] Kalita P., Chakrabarti S., Bhattacharjee B., Paul S., Dutta P.P., Pachuau L. (2025). Recent progress in improving delivery, bioavailability and bioactivity of polyphenolic compounds through encapsulation: A comprehensive review. Food Chem..

[53] Cheng J., Velez F.J., Singh P., Cui L. (2024). Fabrication, characterization, and application of pea protein-based edible film enhanced by oregano essential oil (OEO) micro- or nano-emulsion. Curr. Res. Food Sci..

