# Supplementary material for: Fabrication, Characterization, and Transcriptomic Analysis of Oregano Essential Oil Liposomes for Enhanced Antibacterial Activity and Sustained Release
Source: Foods. 2026 Jan 3;15(1):157. doi: 10.3390/foods15010157 (PMC12786251; doi:10.3390/foods15010157)
Supplement: Supplementary file 1 [file foods-15-00157-s001.zip › foods-4056966-supplementary.pdf]

Supplementary Materials

1. Process optimization of oregano essential oil liposome (OEO-Lip) preparation

1.1 Single-factor experimental design for OEO-Lip preparation

To optimize the preparation conditions of the liposomes, single-factor experiments were conducted to investigate the effects of six factors—egg yolk lecithin to cholesterol mass ratio, OEO concentration, Tween 80 concentration, pH of PBS solution, volume of PBS, and rotary evaporation temperature—on the average particle size, polydispersity index (PDI), and encapsulation efficiency (EE) of the essential oil liposomes. The objective was to identify the optimal level for each factor. All experiments were performed in triplicate. During the optimization process, the following baseline conditions were maintained constant: egg yolk lecithin to cholesterol mass ratio of 4:1 (with egg yolk lecithin fixed at 400 mg), OEO concentration of 2 mg/mL, Tween 80 concentration of 2%, PBS pH of 7.0, PBS volume of 25 mL, and rotary evaporation temperature of 45°C.

Specifically, (1) Using the one-variable-at-a-time method, different egg yolk lecithin to cholesterol mass ratios (1:1, 2:1, 3:1, 4:1, 5:1, 6:1) were tested to evaluate their effects. (2) Different OEO concentrations (1, 1.5, 2, 2.5, 3, 3.5 mg/mL) were tested to evaluate their effects. (3) Different Tween 80 mass fractions (0%, 1%, 2%, 3%, 4%, 5%) were tested to evaluate their effects. (4) PBS solutions with different pH values (5.5, 6.0, 6.5, 7.0, 7.5, 8.0) were tested to evaluate their effects. (5) Different volumes

of PBS (10, 15, 20, 25, 30, 35 mL) were tested to evaluate their effects. (6) Different rotary evaporation temperatures (30, 35, 40, 45, 50, 55 °C) were tested to evaluate their effects.

1.2 Response surface methodology (RSM) experimental design for OEO-Lip preparation

Based on the results of the single-factor experiments, four factors—(A) OEO concentration, (B) PBS volume, (C) rotary evaporation temperature, and (D) egg yolk lecithin to cholesterol mass ratio—were selected as independent variables for the RSM optimization. EE (Y) was chosen as the response variable. A Box-Behnken design (BBD) was employed to optimize the preparation process of OEO-Lip. The factors and their corresponding levels are presented in Table S1-1.

Table S1-1. Response surface test factors and levels

Factor	Level		
	-1	0	1
A: OEO concentration (mg/mL)	A_1	A_2	A_3
B: PBS volume (mL)	B_1	B_2	B_3
C: Rotary evaporation temperature (°C)	C_1	C_2	C_3
D: Lecithin to cholesterol mass ratio	D_1	D_2	D_3

2. Results of optimized preparation of OEO-Lip

2.1 Results of single-factor experiment

As shown in Figure S1(A), the encapsulation efficiency of OEO increased initially and then decreased with rising ratio of egg yolk lecithin to cholesterol, reaching a maximum of 72.87% at a mass ratio of 4:1. Figure S1(B) indicates that the highest

encapsulation efficiency (69.36%) was achieved at an OEO concentration of 2 mg/mL. In Figure S1(C), with increasing Tween 80 content, the average particle size of OEO-Lip decreased, while the encapsulation efficiency first increased and then declined, peaking at 70.7% with a particle size of 155.4 nm when 2% Tween 80 was added. Figure S1(D) demonstrates that at pH 7.0 in PBS solution, the encapsulation efficiency reached its maximum (65.2%) with a minimal average particle size of 149.3 nm. According to Figure S1(E), the highest encapsulation efficiency (71.96%) was observed at a PBS volume of 25 mL. Finally, Figure S1(F) shows that a rotary evaporation temperature of 45 °C resulted in an optimal encapsulation efficiency of 69.77% and a maximum average particle size of 164.4 nm.

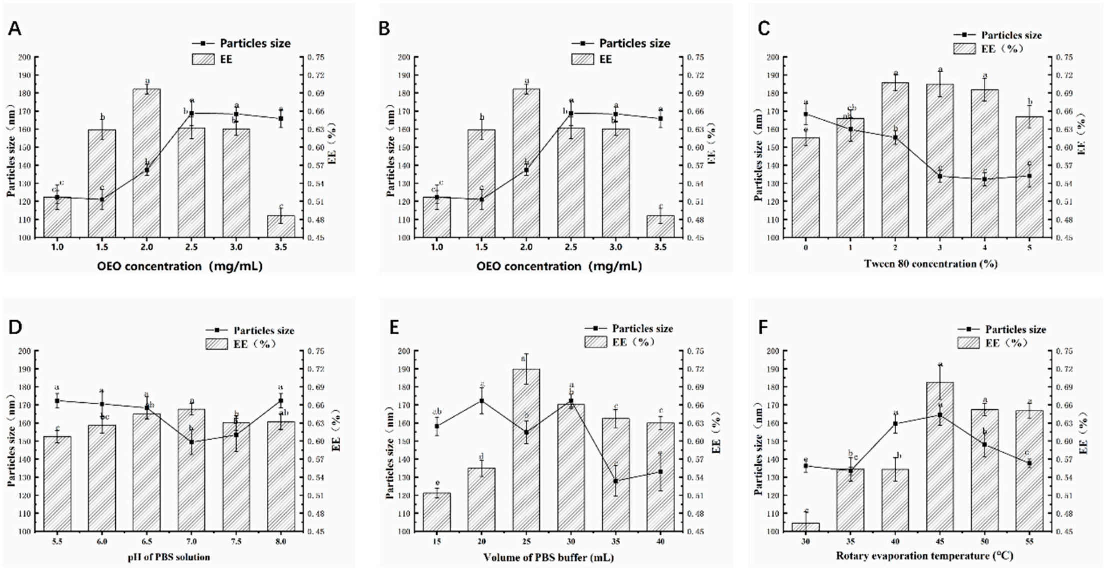

Figure S1. Results of single-factor experiment. (**A**) Lecithin to cholesterol mass ratio. (**B**) OEO concentration. (**C**) Tween 80 concentration. (**D**) pH of PBS solution. (**E**) Volume of PBS buffer. (**F**) Rotary evaporation temperature. a–c indicated the results of one-way ANOVA for comparisons.

2.2 Results of optimization by response surface methodology

Based on the single-factor experiments, four factors with the greatest impact on the liposomal encapsulation efficiency—essential oil concentration (A), PBS volume (B), rotary evaporation temperature (C), and the mass ratio of egg yolk lecithin to cholesterol (D)—were selected for response surface methodology (RSM). The encapsulation efficiency (Y) was used as the response value. Regression analysis was performed using Design-Expert software, and the coded factors and levels are presented in Table S1-2.

Table S1-2. Response surface test factors and levels

Factor	Level		
	-1	0	1
A: OEO concentration (mg/mL)	1.5	2	2.5
B: PBS volume (mL)	20	25	30
C: Rotary evaporation temperature (°C)	40	45	50
D: Lecithin to cholesterol mass ratio	3:1	4:1	5:1

The regression analysis of the relationship between encapsulation efficiency and various factors was performed using Design-Expert software, and the results of the response surface optimization experiments are presented in Table S1-3. The multiple regression equation derived from the regression fitting is as follows:

$$Y = 78.15 - 2.37A + 3.43B - 1.63C - 1.56D + 4.17AB + 2.76AC - 1.37AD - 2.3BC + 1.41BD - 4.51CD - 5.16A^2 - 6.19B^2 - 8.92C^2 - 4.34D^2$$

Table S1-3. Response surface optimization test results

Number	A	B	C	D	Y (EE%)
1	2.5	20	45	4	58.646
2	1.5	25	45	5	70.8376
3	2.5	25	45	5	62.0951

4	2	25	50	3	69.4484
5	2	25	40	3	63.301
6	2	30	45	3	70.305
7	2	25	45	4	78.6404
8	2	25	45	4	78.226
9	1.5	25	45	3	72.7339
10	2.5	25	40	4	60.3301
11	2	20	50	4	60.1004
12	2	20	45	3	66.447
13	2	30	50	4	63.5821
14	2	25	45	4	78.5858
15	2	20	40	4	58.1506
16	1.5	20	45	4	69.7236
17	2.5	25	45	3	69.4807
18	1.5	25	50	4	61.5153
19	2	25	50	5	58.0105
20	2.5	25	50	4	61.5785
21	2	25	45	4	77.066
22	2.5	30	45	4	72.7601
23	1.5	25	40	4	71.3209
24	2	25	40	5	69.8913
25	1.5	30	45	4	67.1718
26	2	25	45	4	78.2511
27	2	20	45	5	61.3498
28	2	30	40	4	70.8464
29	2	30	45	5	70.8611

Based on the equations in Table S1-4, the model was highly significant ($P < 0.0001$), while the lack-of-fit term was not significant ($P > 0.05$). The multiple quadratic regression equation exhibited a correlation coefficient above 0.95, indicating that the fitted regression model can be used to predict the response value and is statistically meaningful. Furthermore, A, B, C, D, AB, AC, BC, CD, A^2, B^2, C^2, and D^2 had highly significant effects on Y ($P < 0.01$), while AD and BD showed significant influences ($P < 0.05$). Response surface plots illustrating the interactions of factors significantly

affecting the encapsulation efficiency are presented in Figure S2. The results indicated that the order of factors influencing encapsulation efficiency was PBS volume > oregano essential oil concentration > rotary evaporation temperature > mass ratio of lecithin to cholesterol, with PBS volume being the most influential factor.

Table S1-4. Response surface analysis of variance

	Sum of squares	Degrees of freedom	Mean square	F-value	P-value
Model	1208.91	14	86.35	69.5	< 0.0001
A	67.27	1	67.27	54.15	< 0.0001
B	140.83	1	140.83	113.35	< 0.0001
C	32.03	1	32.03	25.78	0.0002
D	29.05	1	29.05	23.38	0.0003
AB	69.44	1	69.44	55.89	< 0.0001
AC	30.55	1	30.55	24.59	0.0002
AD	7.53	1	7.53	6.06	0.0274
BC	21.22	1	21.22	17.08	0.001
BD	7.99	1	7.99	6.43	0.0238
CD	81.25	1	81.25	65.4	< 0.0001
A^2	172.46	1	172.46	138.81	< 0.0001
B^2	248.34	1	248.34	199.89	< 0.0001
C^2	516.24	1	516.24	415.51	< 0.0001
D^2	121.92	1	121.92	98.13	< 0.0001
Residual	17.39	14	1.24		
Lack of Fit	15.77	10	1.58	3.89	0.1012
Pure Error	1.62	4	0.41		
Cor Total	1226.3	28			
R^2	0.9858				
Adj-R^2	0.9716				

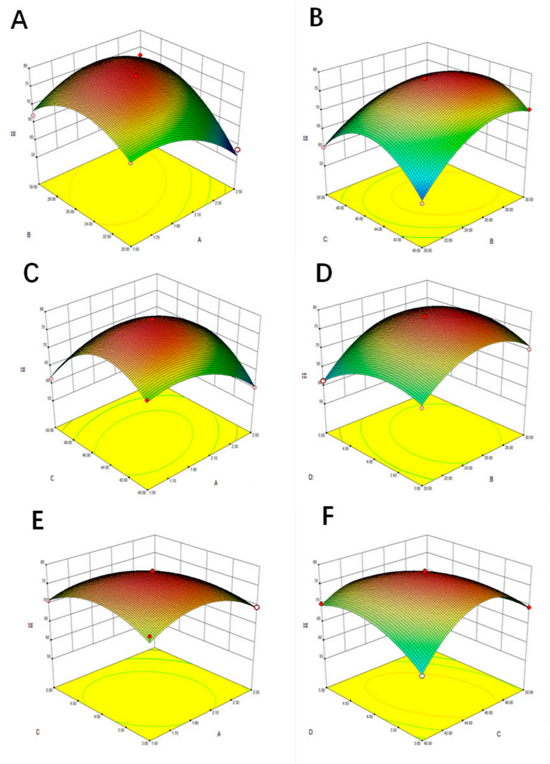

Figure S2. Influence of interaction among factors on embedding rate. (**A**) A: OEO concentration and B: PBS volume; (**B**) B: PBS volume and C: Rotary evaporation temperature; (**C**) A: OEO concentration and C: Rotary evaporation temperature; (D) B: PBS volume and D: Lecithin to cholesterol mass ratio; (**E**) A: OEO concentration and D: Lecithin to cholesterol mass ratio; (**F**) C: Rotary evaporation temperature and D: Lecithin to cholesterol mass ratio.

Based on the results of the above experimental model, the optimal preparation conditions for OEO-Lip predicted by the regression model were as follows: lecithin-to-cholesterol mass ratio of 3.96:1 (lecithin 400 mg, cholesterol 101.01 mg), OEO concentration of 1.82 mg/mL, PBS pH 7.0, PBS volume of 26 mL, and rotary evaporation temperature of 44.34 °C. Under these conditions, the theoretical encapsulation efficiency was predicted to be 78.73%. Three independent validation

experiments yielded an average encapsulation efficiency of 77.77%, with a relative error of 1.22% compared to the theoretical value, indicating a well-fitted model.
